# Characterization of a pleiotropic regulator MtrA in *Streptomyces avermitilis* controlling avermectin production and morphological differentiation

**DOI:** 10.1186/s12934-024-02331-2

**Published:** 2024-04-08

**Authors:** Jinpin Tian, Yue Li, Chuanbo Zhang, Jianyu Su, Wenyu Lu

**Affiliations:** 1 School of Chemical Engineering and Technology, Tianjin University, Tianjin, People’s Republic of China; 2 Frontiers Science Center for Synthetic Biology, Tianjin University, Tianjin, People’s Republic of China; 3 Key Laboratory of System Bioengineering (Tianjin University), Ministry of Education, Tianjin, People’s Republic of China; 4 Key Laboratory of the Ministry of Education for Conservation and Utilization of Special Biological Resources in the Western, Yinchuan, 750021 China; 5 College of Life Science, Ningxia University, Yinchuan, 750021 Ningxia China

**Keywords:** MtrA, Avermectin, *S. avermitilis*, Transcriptional regulators, Secondary metabolism

## Abstract

**Background:**

The macrolide antibiotic avermectin, a natural product derived from *Streptomyces avermitilis*, finds extensive applications in agriculture, animal husbandry and medicine. The *mtrA* (*sav_5063*) gene functions as a transcriptional regulator belonging to the OmpR family. As a pleiotropic regulator, *mtrA* not only influences the growth, development, and morphological differentiation of strains but also modulates genes associated with primary metabolism. However, the regulatory role of MtrA in avermectin biosynthesis remains to be elucidated.

**Results:**

In this study, we demonstrated that MtrA, a novel OmpR-family transcriptional regulator in *S. avermitilis*, exerts global regulator effects by negatively regulating avermectin biosynthesis and cell growth while positively controlling morphological differentiation. The deletion of the *mtrA* gene resulted in an increase in avermectin production, accompanied by a reduction in biomass and a delay in the formation of aerial hyphae and spores. The Electrophoretic Mobility Shift Assay (EMSA) revealed that MtrA exhibited binding affinity towards the upstream region of *aveR*, the intergenic region between *aveA1* and *aveA2* genes, as well as the upstream region of *aveBVIII* in vitro. These findings suggest that MtrA exerts a negative regulatory effect on avermectin biosynthesis by modulating the expression of avermectin biosynthesis cluster genes. Transcriptome sequencing and fluorescence quantitative PCR analysis showed that *mtrA* deletion increased the transcript levels of the cluster genes *aveR*, *aveA1*, *aveA2*, *aveC*, *aveE*, *aveA4* and *orf-1*, which explains the observed increase in avermectin production in the knockout strain. Furthermore, our findings demonstrate that MtrA positively regulates the cell division and differentiation genes *bldM* and *ssgC*, while exerting a negative regulatory effect on *bldD*, thereby modulating the primary metabolic processes associated with cell division, differentiation and growth in *S. avermitilis*, consequently impacting avermectin biosynthesis.

**Conclusions:**

In this study, we investigated the negative regulatory effect of the global regulator MtrA on avermectin biosynthesis and its effects on morphological differentiation and cell growth, and elucidated its transcriptional regulatory mechanism. Our findings indicate that MtrA plays crucial roles not only in the biosynthesis of avermectin but also in coordinating intricate physiological processes in *S. avermitilis.* These findings provide insights into the synthesis of avermectin and shed light on the primary and secondary metabolism of *S. avermitilis* mediated by OmpR-family regulators.

**Supplementary Information:**

The online version contains supplementary material available at 10.1186/s12934-024-02331-2.

## Background

Avermectin is a polyketide antibiotic produced by *Streptomyces avermitilis* which is originally obtained from soil and has significant insecticidal effect on nematodes and arthropods [1]. Furthermore, owing to its unique insecticidal mechanism of action, avermectin has no toxic effect on mammals, while demonstrating potent efficacy against insects that commonly develop resistance to conventional pesticides. Therefore, it is the most commonly used biopesticide in agriculture [2]. The functions of the genes within the avermectin biosynthetic gene cluster and their corresponding biosynthetic pathways have been comprehensively elucidated [3]. *Streptomyces* species are saprophytic, aerobic heterotrophic bacteria that undergo morphological changes from trophic hyphae to spores under complex and variable survival conditions. This transition is accompanied by the production of secondary metabolites and secretion of extracellular enzymes. All these processes are tightly regulated at multiple levels by a variety of transcriptional regulators [4, 5]. In the biosynthesis of secondary metabolites in *Streptomyces*, there are typically two types of regulators. Firstly, pathway-specific regulators, which are generally localized within a specific biosynthetic gene cluster and exert significant influence on the expression level of that particular biosynthetic gene cluster. Secondly, global regulators, also known as pleiotropic regulators, generally reside outside the biosynthetic gene cluster and play a pivotal role in regulating both primary and secondary metabolism [6, 7].

MtrA is a responsive regulatory factor in the two-component system MtrAB, which belongs to the OmpR family and contains a conserved DNA binding domain (HTH), while MtrB encodes a membrane protein. MtrB autophosphorylates its own histidine residue in response to extracellular signals, subsequently phosphorylating the aspartic acid residue of MtrA to induce conformational changes in its DNA-binding domain, thereby modulating the expression of target genes [8–11]. The MtrA gene was initially discovered in *Mycobacterium tuberculosis*, exhibiting a high similarity to the typical response regulators AfsQ1, PhoB, PhoP, and OmpR [12]. The subsequent studies revealed a high degree of conservation of MtrA in the genomes of *Corynebacterium glutamicum* and *M. tuberculosis*. The transcriptional regulator MtrA is indispensable for the survival of *M. tuberculosis* [13], while it does not play a crucial role in the viability of *C. glutamicum*. However, the *mtrA* deficiency in *C. glutamicum* strain has a significant influence on cell morphology, antibiotic sensitivity, and the expression of genes associated with osmotic protection when compared to wild-type strains [14]. These results suggest that MtrA plays an important regulatory role in cell growth and metabolism.

MtrA also plays a conserved role in the regulation of genes related to development and morphological differentiation in *Streptomyces*. In *Streptomyces coelicolor*, MtrA is indispensable for the development of aerial mycelium, with mutant strains exhibiting a distinct bald phenotype [15, 16]. In *Streptomyces venezuela*, MtrA binds to the upstream sequences of the *dnaA* and *dnaN* genes involved in DNA replication, as well as the promoter sequences upstream of the cytokines *ftsZ* and *ssgB*, thereby exerting regulatory control over growth and development processes in *S. venezuela* [17]. The MtrA protein of *Saccharopolyspora erythraea E3* strain, a high erythromycin-producing strain, had a two amino acid (H197 and V198) deletion in the DNA recognition helices within its C-terminal domain, leading to a phenotypic alteration [18].

MtrA exerts a profound influence not only on the growth, development, and morphological differentiation of *Streptomyces* strains but also on genes associated with primary metabolism. The growth and development of *Streptomyces* cells, as well as the synthesis of secondary metabolites, require a balanced supply of carbon, nitrogen, and phosphorus. Among these elements, glutamate and glutamine serve as the primary intracellular nitrogen donors [19]. Zhu et al. demonstrated for the first time that MtrA is a new regulator of nitrogen metabolism. In *S. coelicolor*, MtrA and GlnR recognize similar conserved sequences. MtrA represses the expression of nitrogen metabolism-related genes such as *glnII*, *nirB*, and *ureA*, in a nitrogen-rich environment, which is in contrast to the regulatory effect of GlnR. It was also found that *glnR* itself is a direct target of MtrA, and MtrA inhibits the transcription of *glnR* [8]. In the following study, Zhu et al. further proved that both MtrA and GlnR exhibit binding affinity for the upstream region of nitrogen metabolism genes, thereby suggesting a competitive interaction between these two transcription factors. MtrA binds more strongly to nitrogen metabolism genes under nitrogen-rich conditions, while GlnR binds more strongly to these genes under nitrogen-limited conditions. Additionally, both MtrA and GlnR possess the ability to regulate their own gene expression [9].

As a global regulator, MtrA positively or negatively regulates antibiotic production in a wide range of *Streptomyces* species and also has the potential to activate clusters of silent genes. However, the regulation of MtrA metabolic network in *Streptomyces* is still limited to a few model strains such as *S. coelicolor* and *S. venezuelae*. Moreover, the specific targets and regulatory mechanisms of MtrA in *Streptomyces* are not yet fully elucidated, necessitating in-depth exploration of the MtrA regulatory network, which will facilitate a comprehensive understanding, ultimately leading to the development of enhanced *Streptomyces* strains with heightened antibiotic production.

In this study, we investigated the regulatory role and mechanism of MtrA on avermectin biosynthesis through molecular biology modification, EMSA, fluorescence quantitative PCR and transcriptome sequencing. These findings contribute to a deeper understanding of the primary and secondary metabolism regulatory network in *S. avermitilis*, which holds significant implications for enhancing industrial production of avermectin.

## Results

### Characterization of MtrA and its adjacent genes

**Fig. 1:**
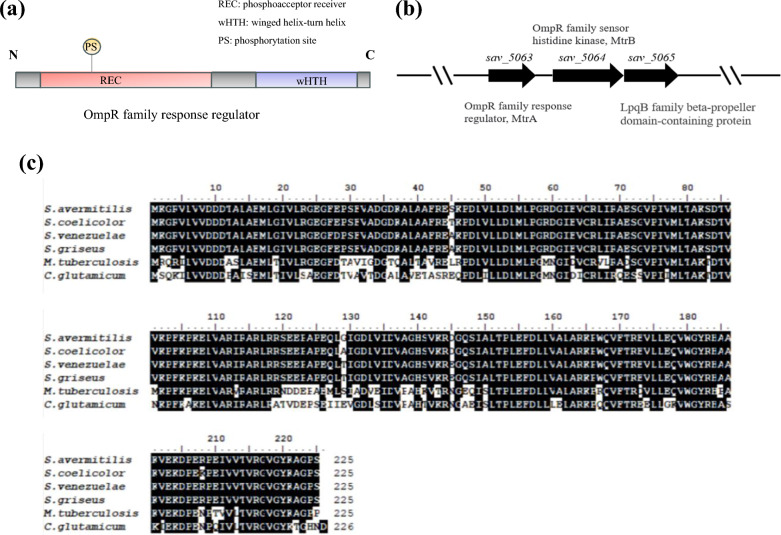
Bioinformatics analysis of MtrA in *S. avermitilis*. **a** Structural characterization of the OmpR family. **b** *mtrA* and nearby genes. **c** Homologous proteins of MtrA in *S. avermitilis*, *S. coelicolor*, *S. venezuelanus*, *S. griseus*, *M. tuberculosis* and *C. glutamicum*

MtrA, a response regulator in the MtrAB two-component system, belongs to the OmpR family, which contains the signal receptor REC and the wHTH structural domain [20] (Fig. 1a). In order to study the function of MtrA in *S. avermitilis*, we performed a BLAST analysis using the amino acid sequence of MtrA from *S. coelicolor* as a query to identify its homologous sequence *sav_5063* (GenBank: BAC72775.1) in *S. avermitilis*, revealing a protein sequence similarity of 94%. The sequence of *mtrA* gene in *S. avermitilis* is 690 bp long and encodes a protein consisting of 225 amino acids. *mtrB* is located downstream of *mtrA*, with a total length of 2079 bp, encoding a protein comprising 692 amino acids. *mtrB* is a homologous sensor kinase gene of *mtrA*, which regulates the phosphorylation state of *mtrA* and *lpqB*. The *lpqB* gene, which is immediately adjacent to *mtrB*, encodes a lipoprotein (Fig. 1b). Multiple sequence alignment analysis of MtrA in different *Streptomyces* revealed a remarkable level of conservation, indicating the pivotal biological functions played by MtrA and its homologous proteins in *Streptomyces* (Fig. 1c).

### MtrA affects avermectin production, cell growth and morphological differentiation

**Fig. 2:**
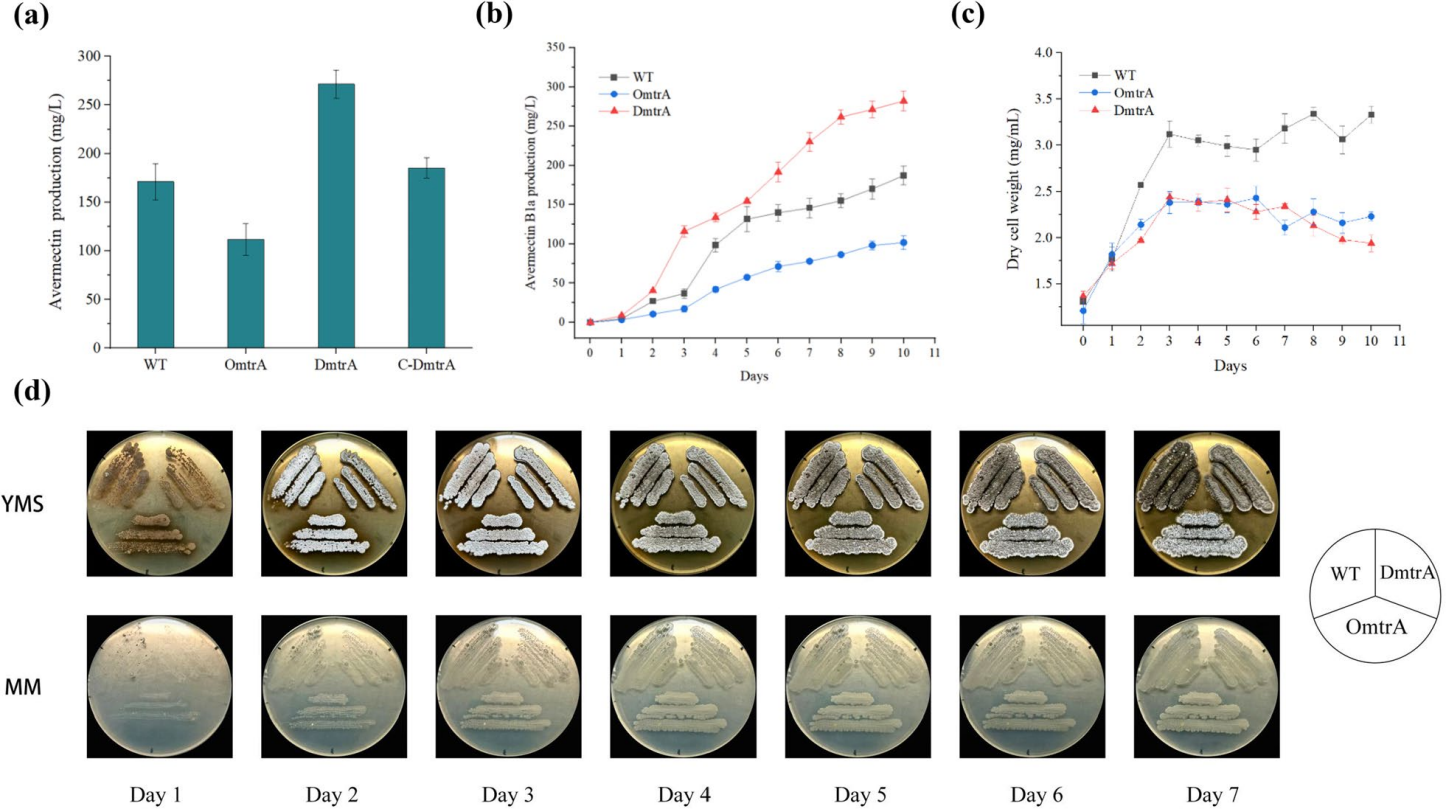
Effects of *mtrA* deletion and overexpression on avermectin production, cell growth and morphological differentiation.** a** Avermectin production by *mtrA*-related strains WT: wild-type strain, OmtrA: *mtrA* overexpressing strain, DmtrA: *mtrA* deletion strain, C-DmtrA: *mtrA* complementary strain. **b** Production of avermectin curve of WT, OmtrA and DmtrA strains at different time points. **c** Dry cell weight of WT, OmtrA and DmtrA strains at different time points. **d** Growth patterns of *mtrA*-related strains at different medium

In order to study the effects of MtrA on avermectin biosynthesis, the gene deletion strain DmtrA, the overexpression strain OmtrA, and the complementation strain C-DmtrA were constructed and then fermented them in shake flasks. As shown in Fig. 2a, avermectin production decreased by 34.68% in the OmtrA strain and increased by 58.68% in the DmtrA strain compared to the wild strain. Moreover, the production of avermectin by C-DmtrA was restored to the level observed in the wild-type strain. These results suggest that MtrA may negatively regulates avermectin biosynthesis in *S. avermitilis*.

In order to investigate the changes of avermectin production during the fermentation process, samples were collected daily at consistent time points, and the avermectin production of the WT, OmtrA, and DmtrA strains was quantified (Fig. 2b). During a 10 day fermentation period, the production of avermectin among the three strains exhibited similar trends. The synthesis of avermectin started with the second day and demonstrated rapid accumulation from days 2 to 10, ultimately reaching maximum yield by the tenth day. The production of avermectin in the DmtrA strain consistently increased compared to that in the wild-type strain starting from day 2. Conversely, it decreased from day 2 onwards in the overexpression strain OmtrA.

As shown in Fig. 2c, the biomass of the three strains exhibited a consistent trend throughout the fermentation cycle. Specifically, they underwent logarithmic growth during the initial 3 days and subsequently transitioned into a stable phase. However, the biomass of OmtrA and DmtrA was similar throughout the growth cycle and both were reduced compared to the wild-type. The findings demonstrated that MtrA exerts a dual regulatory role by suppressing avermectin biosynthesis and impacting mycobacterial growth. Moreover, the variation in avermectin production may be partially attributed to the influence exerted by bacterial biomass.

The growth morphology of the engineered strains was then observed on YMS and MM plates, respectively (Fig. 2d). DmtrA exhibited delayed formation of aerial hyphae on YMS plates compared to WT and OmtrA, while the production of gray spores by DmtrA was slightly postponed. On the 7th day, WT and DmtrA formed dense spores. However, the spores formed by WT appeared darker in color, while those formed by DmtrA exhibited a more pronounced gray color. The OmtrA spores exhibit a combination of black and white pigmentation, with a predominant presence of white spores. The three strains grown on MM plates did not show significant differences in growth morphology (Fig. 2d).

In summary, these findings suggest that MtrA acts as a global regulator, exerting negative control over avermectin biosynthesis and influencing mycobacterial growth and morphological differentiation.

### Binding of MtrA to promoters within the avermectin biosynthesis gene cluster

**Fig. 3:**
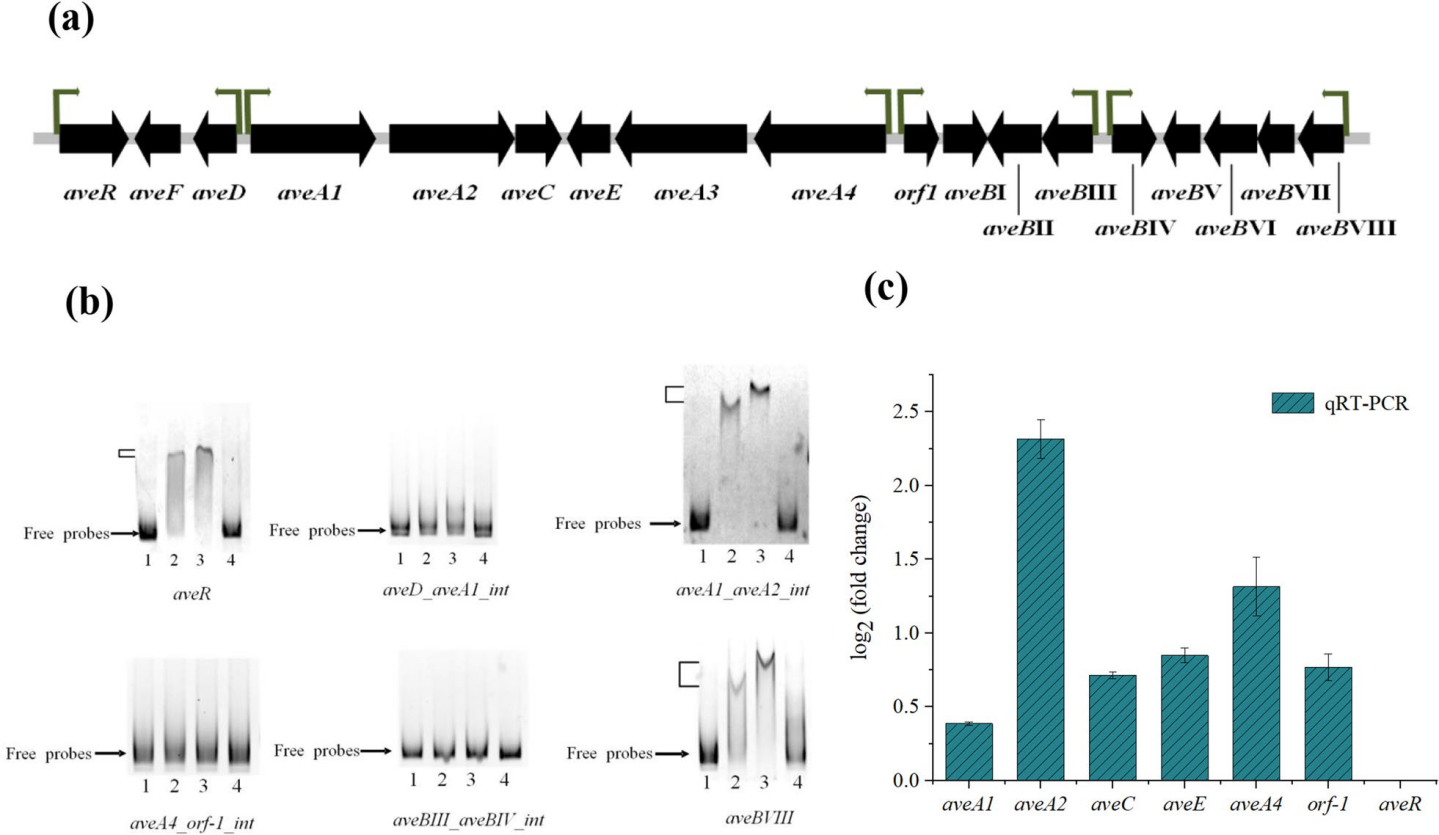
Binding and transcriptional analysis of MtrA with avermectin biosynthesis related genes.** a** Predicted promoters in the avermectin biosynthesis gene cluster. **b** EMSA analysis of the binding of MtrA to the promoter of avermectin synthesis related genes (lane 1: 50 ng labeled probe, lane 2: 50 ng labeled probe + 3 μg MtrA protein, lane 3: 50 ng labeled probe + 6 μg MtrA protein, lane 4: 50 ng labeled probe + 3 μg MtrA protein + excess unlabeled competing probes). **c** qRT-PCR results for genes *aveA1*, *aveA2*, *aveC*, *aveE*, *aveA4*, *orf-1*, and *aveR* within the avermectin biosynthesis cluster. qRT- PCR was performed using *S. avermitilis* 16S *rRNA* as a reference gene, resulting in the transcript levels of each gene compared to the reference gene

Transcriptional regulators typically bind to conserved motifs in the promoter region of target genes. Therefore, possible promoters within the avermectin biosynthetic gene cluster were initially predicted. The upstream region of the cluster genes was predicted to contain six potential conserved motifs, including an intergenic region between *aveA1* and *aveA2* (Fig. 3a). The ability of MtrA to bind these promoter regions was assessed by EMSA. The results showed that MtrA could directly bind to the promoter region of the *aveA1* and *aveA2* gene intervals, as well as *aveR* and *aveBVIII* (Fig. 3b).

The *aveR* gene encodes a pathway-specific activator essential for the expression of all avermectin biosynthesis genes [5, 21, 22]. To investigate whether MtrA regulates avermectin production through pathway-specific activators, we performed qRT-PCR validation to explore changes in the expression levels of avermectin biosynthesis-related genes in both wild-type and *mtrA* deletion strains (Fig. 3c). The qRT-PCR analysis revealed varying degrees of up-regulation in the transcript levels of *aveA1*, *aveA2*, *aveC*, *aveE*, *aveA4*, and *orf-1* genes. Notably, the expression of the *aveA2* gene showed an approximately 2.3-fold increase in transcript level. Therefore, it is hypothesized that the deletion of *mtrA* deregulates the negative regulatory effect on *aveA2*, resulting in an increase in avermectin production. This alteration also influences the transcript levels of other genes within the cluster.

### Binding of MtrA with genes related to cell growth and morphological differentiation

**Fig. 4:**
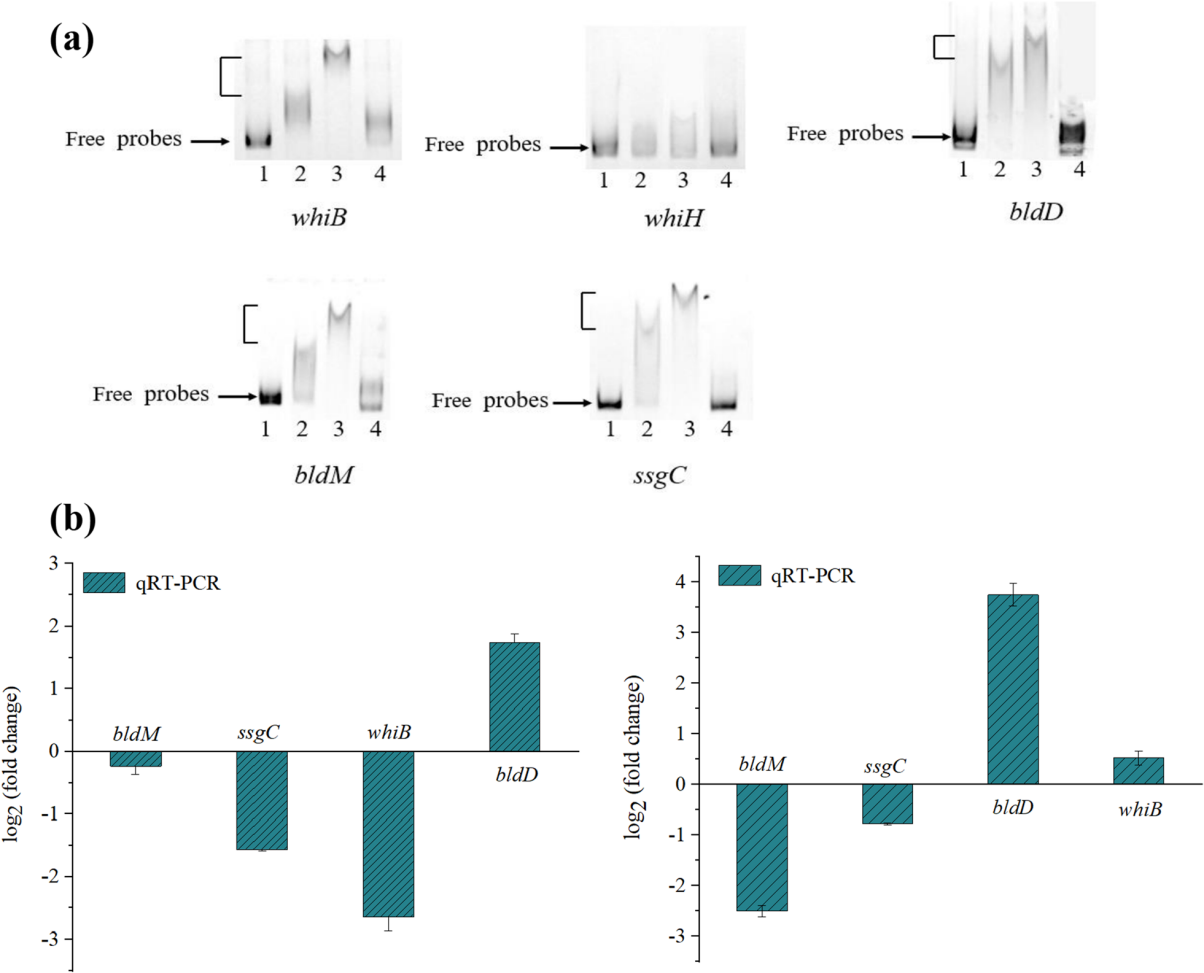
Predicted target genes involved in cell growth and morphological differentiation. **a** EMSA analysis of MtrA binding to promoters of morphological differentiation related genes (lane 1: 50 ng labeled probe, lane 2: 50 ng labeled probe + 3 μg MtrA protein, lane 3: 50 ng labeled probe + 6 μg MtrA protein, lane 4: 50 ng labeled probe + 3 μg MtrA protein + excess unlabeled competing probes). **b** qRT-PCR validation of day 2 (left) and day 6 (right) fermentation genes *bldM*, *ssgC*, *whiB* and *bldD*. qRT- PCR used 16S *rRNA* as a reference gene to obtain the transcript levels of individual genes relative to the reference gene

A conserved binding sequence of 14 bp of MtrA (CRTCGRYGACAAGG, where R represents A or G and Y represents C or T) was obtained using MEME the website prediction (Additional file 1: Fig. S5). The conserved binding sequences obtained were then input into FIMO to predict potential binding sites in the genes of *S. avermitilis*. We performed EMSAs on several putative MtrA targets involved in cell growth and morphological differentiation: *whiB* (*sav_5042*, encoding a WhiB-family transcriptional regulator essential for sporulation), *whiH* (*sav_2445*, putative spore transcriptional regulators), *bldD* (*sav_6861*, encoding a DNA-binding protein), *bldM* (*sav_4998*, encoding a two-component system response regulator), *ssgC* (*sav_6810*, encoding a putative cell division protein). The results showed that the MtrA protein bound to the promoter regions of *whiB*, *bldD*, *bldM* and *ss*gC genes, but not to the promoter region of *whiH* gene (Fig. 4a). This suggests that MtrA can directly regulate the expression of *whiB*, *bldD*, *bldM* and *ssgC* genes, thereby affecting the growth, development and morphological differentiation of *S. avermitilis*.

Transcription levels of *whiB*、*bldD*、*bldM* and *ssgC* genes were determined by qRT-PCR in WT and DmtrA strains (Fig. 4b). At both day 2 and day 6 of fermentation, the transcription levels of *bldM* and *ssgC* exhibited down-regulation, while *bldD* showed up-regulation, suggesting that MtrA can differentially regulate the expression of these genes. The down-regulation of *ssgC* transcription levels affected the normal cell growth and division of *S. avermitilis*, potentially accounting for the lower biomass observed in the DmtrA strain compared to the WT strain throughout the fermentation process. MtrA exerts a negative regulatory effect on *bldD* expression, and the deletion of this gene resulted in an elevated transcript level of *bldD*. This result was consistent with previous findings reported in *S. coelicolor* [16]. However, *mtrA* deletion did not cause *S. avermitilis* to develop a “*bald*” phenotype, suggesting that the absence of *mtrA* does not affect the formation of gray spores in this organism.

### Transcriptome analysis of MtrA-deletion strains

**Fig. 5:**
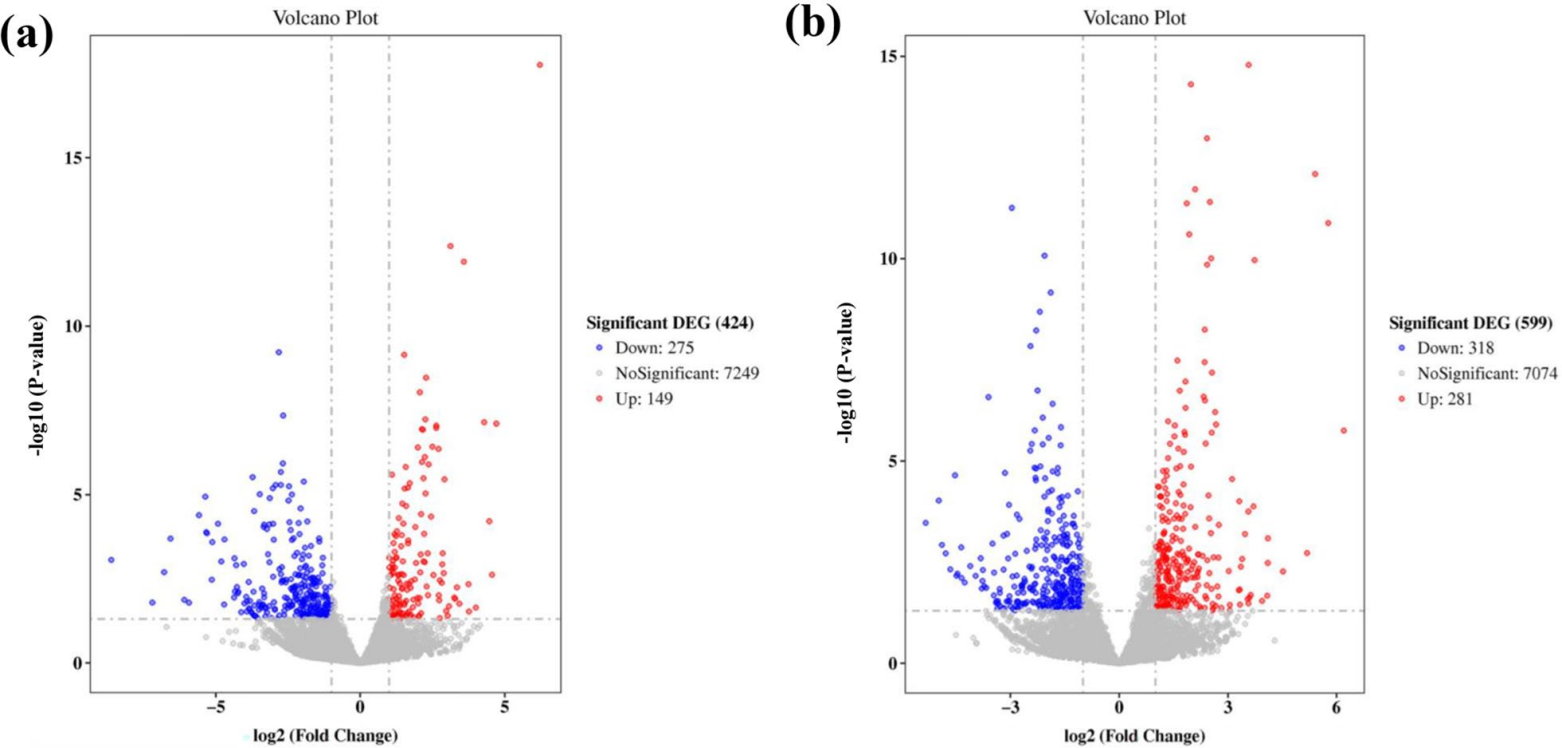
Volcano plots of MtrA regulated gene on day 2 of fermentation **(a)** and volcano map on day 6 **(b)**

Fermentation broth samples of WT and DmtrA strains were collected on the second day, representing the logarithmic phase of growth and early stage of avermectin synthesis, as well as on the sixth day, corresponding to the stable phase of growth. These samples were subsequently subjected to RNA-seq analysis. The results revealed that a total of 149 genes were significantly up-regulated and 275 genes were significantly down-regulated in the DmtrA strain at day 2 compared to the wild-type strain. Furthermore, a total of 281 genes exhibited significant up-regulation, while 318 genes displayed significant down-regulation on day 6 (Fig. 5). These differentially expressed genes (DEGs) play crucial roles in a wide range of primary and secondary metabolism pathways within *Streptomyces*.

The precursors involved in avermectin biosynthesis encompass the initial units isobutyryl-CoA and 2-methylbutyryl-CoA, along with the extension units methylmalonyl-CoA and malonyl-CoA. The analysis of transcriptome DEGs revealed a trend towards up-regulation of several crucial primary metabolic pathway genes related to precursor synthesis on days 2 and 6 of fermentation, including *accD1* (*sav_5278*, encoding the beta subunit of acetyl/propionyl coenzyme A carboxylase), *acsA3* (*sav_562*, encoding acetyl-CoA synthase), *ccrA1* (*sav_2890*, encoding crotonyl-CoA reductase), *ccrA2* (*sav_1911*, encoding crotonyl-CoA reductase), *fadE24* (*sav_4390*, encoding acetyl-CoA dehydrogenase), *fadE28* (*sav_4210*, encoding acetyl-CoA dehydrogenase), *fabH7* (*sav_2276*, encoding 3-oxoacyl-ACP synthetase III), *sav_1553* (encoding methyltransferase). This suggests that *mtrA* deletion increased the expression levels of genes associated with avermectin precursor biosynthesis, thereby promoting avermectin biosynthesis. In addition, *mtrA* gene deletion affected the transcription of other transcriptional regulators (Additional file 1: Table S2).

The GO enrichment analysis revealed that the DEGs were enriched in terms related to “structural components of ribosomes”, “rRNA binding” and “ribosomes”. These findings suggest that compared to the WT strain, DmtrA exhibit substantial alterations in both ribosome production and function. The enrichment of “NADH dehydrogenase activity” and “respiratory chain” implies significant alterations in the energy metabolism of the cell. Differential genes are enriched in “catalytic activity”, “translation” and “arginine biosynthesis process”, indicating that the cellular system also undergoes significant alterations in amino acid and protein synthesis (see Additional file 1: Fig. S6). These findings suggest that MtrA may be implicated in the regulation of fundamental cellular metabolic processes, thereby playing a pivotal role as a global regulator. The KEGG analysis was also perfomed and the results were presented in Additional file 1: Fig. S7. The DEGs on day 2 were mainly enriched in the pathways of “secondary metabolite biosynthesis” and “antibiotic biosynthesis”, implying that genes related to antibiotic biosynthesis may exhibit earlier or enhanced transcription in DmtrA. The enrichment analysis of DEGs on day 6 revealed a significant upregulation in the pathways associated with oxidative phosphorylation, amino acid metabolism, and starch and sucrose. The process of oxidative phosphorylation contributes a substantial amount of cellular energy, and the enrichment in “Arginine biosynthesis”, “alanine and aspartate and glutamate metabolism” pathways are observed at both time points (see Additional file 1: Fig. S7). Arginine plays a pivotal role in bacterial protein synthesis and serves as a crucial source of nitrogen, carbon and energy [23]. Glutamic acid can be used as an amino donor in biochemical reactions, and aspartic acid can be synthesized from oxaloacetic acid through an amination reaction, which further produces lysine, homoserine, threonine, and isoleucine. These amino acids may provide precursors for avermectin biosynthesis, potentially contributing to the enhanced antibiotic production observed in later stages.

### DEGs related to central carbon metabolism in *S. avermitilis*

**Fig. 6:**
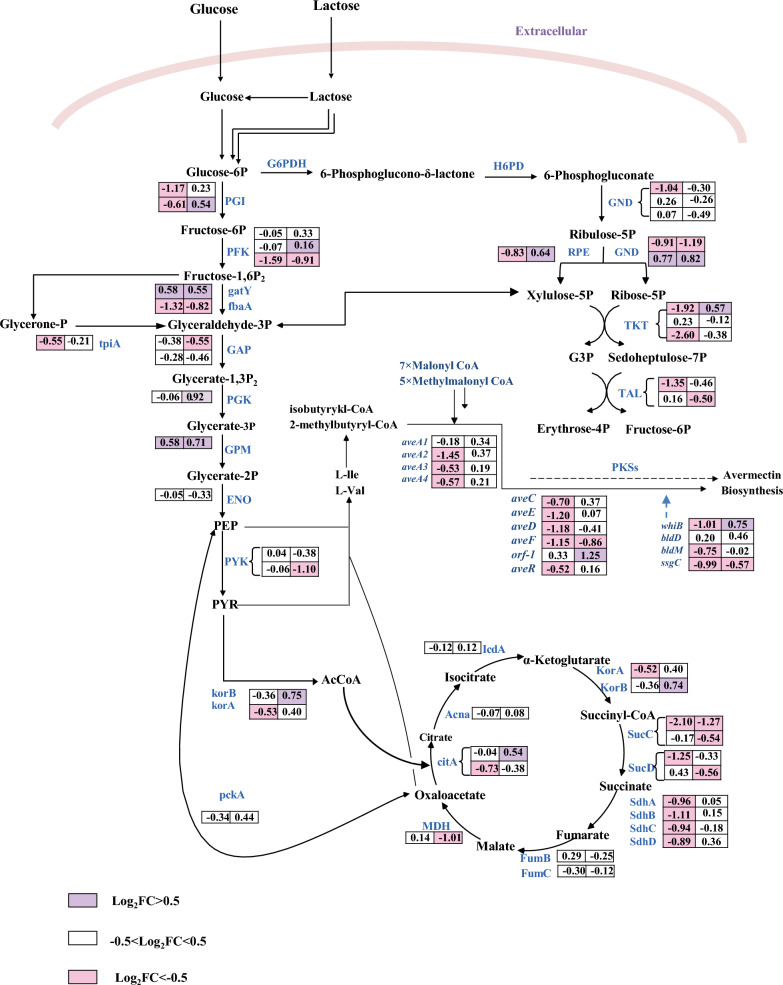
Differential expression genes related to central carbon metabolism in the WT VS DmtrA of *S. avermitilis*

Central carbon metabolism represents a pivotal metabolic network in living organisms, including the glycolysis pathway, the tricarboxylic acid cycle and the pentose phosphate pathway. The DEGs in central carbon metabolism are collected and plotted in Fig. 6. Overall, the carbon metabolism genes in the DmtrA strain are mostly down-regulated, which may explain the reduced biomass resulting from *mtrA* deletion. This finding suggests that MtrA potentially governs the regulation of the central carbon metabolic pathway and the expression of associated carbon metabolism genes, although the precise regulatory mechanism remains uncertain.

## Discussion

The morphological differentiation and secondary metabolism of *Streptomyces* are regulated by a sophisticated regulatory network, enabling adaptation to diverse requirements environments and growth requirements. The MtrAB two-component system (TCS), which is conserved in Actinobacteria, plays multiple roles in cell wall metabolism, cell division, DNA replication, and cell proliferation [24, 25]. MtrB autophosphorylates its own histidine residue in response to extracellular signals, subsequently phosphorylating the aspartic acid residue of MtrA to induce conformational changes in its DNA-binding domain, thereby modulating the expression of target genes [8–11]. Deletion of MtrA or MtrB alters the cellular morphology of *Mycobacterium*. Previous studies using *M. tuberculosis* reported that its MtrA directly regulates cell envelope homeostasis via peptidoglycan synthetase genes *ftsI*, *dacB1*, *wag31*, peptidoglycan hydrolase genes *ripA*, *rpfA-E*, and genes involved in mycolic acids assembly (*fbpB*, *fbpC*). MtrB can interact with peptidoglycan synthetase FtsI, Wag31, and cell envelope biosynthesis regulators PknA and PknB [26, 27]. However, subsequent studies have shown that MtrB is not necessary, indicating that MtrA can be phosphorylated through cross-talk in the absence of its specific sensor kinase [28]. In *S. venezuelae*, MtrA coordinates antibiotic production with sporulation and that deletion of the sensor kinase gene *mtrB* results in constitutively active MtrA and constitutive high-level production of chloramphenicol, as well as a global shift in the metabolome. The deletion of *mtrB* also impacts normal bacterial division, spore production, and antibiotic synthesis [17]. In the present study, we found that the deletion of MtrA in *S. avermitilis* did not result in a significant difference in transcript levels of MtrB (log_2_FoldChange = − 0.24).

The genes responsible for antibiotic synthesis are typically clustered and regulated by cluster-situated regulators (CSRs), which in turn are controlled by various types of higher-level pleiotropic regulators, thus forming a complex regulatory network. The present study demonstrates that MtrA functions as a global regulator governing avermectin production, cell growth, and morphogenesis in *S. avermitilis*. EMSA and qRT-PCR results suggest that MtrA may exert its influence on avermectin production through direct interaction with AveR, the sole CSR identified in the *ave* gene cluster responsible for activating transcription of all *ave* structural genes [4, 5]. Transcriptome sequencing and qRT-PCR analysis showed that *mtrA* deletion increased the transaction levels of the cluster genes *aveR*, *aveA1*, *aveA2*, *aveC*, *aveE*, *aveA4* and *orf-1*. Notably, the transcript level of the *aveA2* gene exhibited an approximately 2.3-fold increase. It is hypothesized that the main reason for the increased avermectin production is that the deletion of *mtrA* deregulates the negative regulatory effect on *ave* cluster genes. Furthermore, the deletion of the *mtrA* gene exerted an impact on the transcriptional activity of various other regulatory factors. SAV4189 is a transcriptional regulator that has been reported to be able to activate avermectin biosynthesis [29].The transcriptome data revealed a significant upregulation of SAV4189 transcription on day 6 of fermentation, implying the potential role of this regulatory factor in facilitating avermectin biosynthesis. The deletion of *mtrA* led to significant alterations in the transcript levels of numerous previously unreported transcriptional regulators (Additional file 1: Table S2), which implied the complex regulatory networks of *mtrA*.

We also observed that MtrA plays a pivotal role in cellular growth and morphological differentiation and have successfully identified five development-associated genes (*whiB*, *bldD*, *bldM* and *ssgC*) that are directly regulated by MtrA. WhiB and WhiA encode transcriptional regulators associated with spore division in *Streptomyces*, as well as proteins essential for cell division in *M. tuberculosis* [30]. The *whi* and *bld* genes constitute a regulatory network that governs development of *Streptomyces,* with BldD serving as a key regulator [31]. Recently Hao Yan et al. have reported the novel finding that BldD_SAV_ can positively regulate abamectin biosynthesis by activating the cluster gene *aveR* [32]. This suggests that BldD not only governs the morphological differentiation of *Streptomyces*, but also exerts regulatory control over of antibiotics production in this genus*. ssgC* encodes a cell division protein that is also essential for spore initiation [33]. Thus, the reduced expression of *bldM* and *ssgC* in DmtrA may be responsible for the delayed formation of aerial mycelium and spores, as well as low biomass. The *mtrA* deletion did not affect gray spore formation in *S. avermitilis*, but the overexpression of *mtrA* resulted in a decrease in gray spores production. The capacity of gray spores to produce avermectin was found to be higher compared to that of white spores. Therefore, revealing the intricate interplay among spore formation, avermectin production, and the regulatory network governed by MtrA necessitates further investigation. The deletion of *mtrA* was observed to result in a reduction in *S. avermitilis* biomass, while leading to an augmentation in avermectin production. In order to get high-performance engineered *S. avermitilis* strains, precise regulation of *mtrA* expression through a fine-tuning promoter could be the key factor in balancing growth and avermectin production in future studies. Additionally, further investigation is warranted to explore the contribution of other putative target genes of MtrA to the observed phenotype of DmtrA.

The starter units 2-methylbutyryl-CoA and isobutyryl-CoA of avermectin biosynthesis are derived from isoleucine and valine, respectively. *acsA3* encodes acetyl coenzyme A synthase, *ccrA1* and *ccrA2* encode crotonyl coenzyme A reductase, *fadE24* and *fadE28* encode acetyl coenzyme A dehydrogenase, *fabH7* encodes 3-oxoacyl-ACP synthetase III, and *sav_1553* encodes methyltransferase. The transcription levels of these genes were all up-regulated, potentially enhancing cellular metabolism to facilitate increased energy production, participating in fatty acid degradation and acetyl coenzyme A synthesis, thereby providing more precursors for avermectin biosynthesis. The elongation of the avermectin polyketide chain necessitates the addition of seven malonyl-CoA units and five methyl malonyl-CoA units to the initial building blocks [34]. The *accD1* gene encodes the beta subunit of acetyl/propionyl coenzyme A carboxylase, which plays a crucial role in catalyzing the conversion of acetyl-CoA to malonyl-CoA. Consequently, the upregulation of *accD1* in DmtrA may enhance the flux of acetyl-CoA towards malonyl-CoA, thereby promoting avermectin biosynthesis. The KEGG enrichment analysis revealed significant enrichment of DEGs in key pathways including oxidative phosphorylation, amino acid metabolism, and ribosome biogenesis, which implied an enhanced utilization of carbon sources to facilitate cellular proliferation or the synthesis of secondary metabolites. However, the biomass exhibited a decrease in DmtrA stain. We hypothesized that the deletion of *mtrA* may lead to the differential expression of genes associated with secondary metabolites. Consequently, the DEGs from additional gene clusters associated with secondary metabolites were collected and depicted in Additional file 1: Table S3. Oligomycin is an antibiotic produced by *S. avermitilis* with important pharmacological activities. Deletion of the *mtrA* gene resulted in alterations in the expression of genes associated with oligomycin biosynthesis, namely *olmA2* and *olmA4*. The up-regulated expression of melanin and lycopene biosynthesis genes (*melC1*, *crtI*, and *crtV*) were also observed in DmtrA strain. Additionally, the up-regulation of certain genes, such as *pks3-2* and *pks9-1*, within unidentified gene clusters implies that the deletion of *mtrA* may induce the expression of silent gene clusters, which may provide targets for the mining of novel secondary metabolites.

**Fig. 7:**
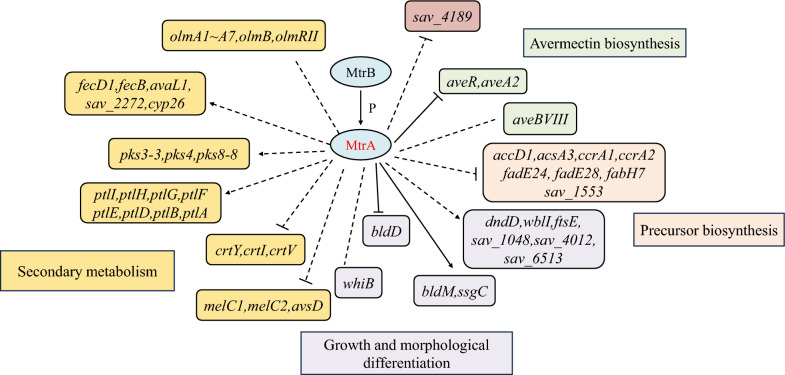
Proposed regulatory model for the regulatory effects of MtrA on avermectin biosynthesis, growth and morphological differentiation and secondary metabolic regulation

Based on the present findings, we propose a model of the MtrA-mediated regulatory network involved in primary metabolism, secondary metabolism, and morphological development (Fig. 7). In this model, MtrA exerts direct control over the expression of avermectin biosynthesis as well as cell morphological differentiation related genes, while also indirectly regulating genes involved in primary metabolism and other secondary metabolic gene clusters, thereby controlling the avermectin biosynthesis, growth, and morphological differentiation of *S. avermitilis*.

## Conclusion

In this study, we focus on elucidating the regulatory effects of the transcriptional regulator MtrA on avermectin biosynthesis in *S. avermitilis*, while also investigating its impact on growth, development, and morphological differentiation of *S. avermitilis*. MtrA was found to act as a global regulator, which negatively regulates avermectin biosynthesis and affects mycobacterial growth and morphological differentiation. The findings further enhance the investigation of the MtrA regulatory network, as well as shed light on the regulation of avermectin biosynthesis.

## Materials and methods

### Plasmids, strains and growth conditions

Plasmids and strains of *E. coli* and *S. avermitilis* used in this study are listed in Table 1. *S. avermitilis* ATCC31267 was used as parent strain for gene disruption and overexpression*. E. coli* strains were cultured in Luria–Bertani (LB) medium at 37 °C. *S. avermitilis* was grown on solid YMS medium at 28 °C for sporulation [35]. YEME liquid medium was used to culture mycelia. Minimal medium (MM) supplemented was used for phenotype observation of *S. avermitilis* [36]*. E. coli* BL21 (DE3) is utilized for MtrA protein expression, while *E. coli* ET12567 is employed for conjugal transfer with *S. avermitilis* [37].

**Table 1:** Strains and plasmids used in this study

Strains/plasmids	Characteristics	Source
*E. coli*		
DH5α	General cloning host for plasmid manipulation	Lab collection
BL21(DE3)	The host of protein overexpression	Lab collection
ET12567/pUZ8002	Strain for conjugal transfer, with pUZ8002 helper plasmids, Chl^R^, Kan^R^	Lab collection
BL21/pET28a-MtrA	E. coli BL21(DE3), with MtrA expression vector pET28a-MtrA, Kan^R^	This work
*S. avermectin*		
ATCC 31267	Wild-type avermectin producer	Lab collection
OmtrA	The wild-type strain with *mtrA* overexpression vector pIBOmtrA, Apr^R^	This work
DmtrA	The wild-type strain with *mtrA* deletion vector pKCDmtrA, Apr^R^	This work
C-DmtrA	The DmtrA strain with *mtrA* complemented vector pSECmtrA, Apr^R^	This work
Plasmids		
pSET152	Integrative *E. coli-streptomyces* shuttle vector, Apr^R^	Lab collection
pIB139	pSET152 derivative carrying *Streptomyces* strong constitutive promoter ermE*p, Apr^R^	Lab collection
pKCcas9dO	Temperature-sensitive replicon pSG5, Apr^R^	Lab collection
pIBOmtrA	*mtrA* overexpression vector based on pIB139	This work
pKCDmtrA	*mtrA* deletion vector based on pKCcas9dO	This work
pSECmtrA	*mtrA* complemented vector based on pSET152	This work
pET-28a ( +)	Vector for protein expression in *E. coli*, N-terminal His_6_-tag, Kan^R^	Novagen

### Gene deletion, complementation, and overexpression

The upstream and downstream homology arms of *mtrA* were obtained by PCR amplification using the wild-type (WT) *S. avermitilis* genome as a template and m-left-F/m-left-R and m-right-F/m-right-R as primers. The obtained fragments were ligated and cloned into pKCcas9dO to obtain the deletion vector pKCDmtrA. The successfully constructed deletion vector pKCDmtrA was transformed into *E. coli* receptor ET12567/pUZ8002 for demethylation. After verifying that the correct single colony was obtained, the construct was transferred into wild-type *S. avermitilis* to obtain the deletion strain DmtrA (Additional file 1: Fig. S1).

The *mtrA* fragment was amplified by PCR using the wild-type *S. avermitilis* genome as a template and O-m-F/O-m-R as primers. The obtained fragment was digested with restriction endonucleases *Nde*I and *EcoR*I and ligated to the downstream of strong promoter ermE*p on plasmid pIB139 which had undergone the same enzymatic treatment to obtain the overexpression vector pIBOmtrA. The successfully constructed pIBOmtrA vector was transformed into ET12567/pUZ8002 for demethylation, and then transferred into wild-type *S. avermitilis* to obtain the overexpression strain OmtrA (Additional file 1: Fig. S2).

Linear fragments of pSET152 were amplified and purified for recovery using pSET-F/pSET-R as primers. The *S. avermitilis* genome was used as a template, and C-m-F/C-m-R were used as primers to amplify a DNA fragment containing MtrA’s own promoter and structural genes and recovered by purification. The fragment was cloned into pSET152 to obtain the complementation vector pSECmtrA. The successfully constructed complementation vector pSECmtrA was introduced into the DmtrA strain in which the deletion vector was discarded by splice transfer, and the correct complementation strain C-DmtrA was screened (Additional file 1: Fig. S3). The primers used in this study are shown in Additional file 1: Table S1.

### Overexpression and purification of MtrA

The wild-type *S. avermitilis* genome was used as a template, and MtrA-F/MtrA-R were used as primers to amplify the *mtrA* gene. Linear fragments of the pET28a vector were obtained by amplification using pET28a-F/pET28a-R as primers. The expression vector pET28a-MtrA was constructed by seamlessly fusing the *mtrA* gene fragment with a linear fragment of the vector using the Seamless Cloning Kit. Subsequently, it was transformed into *E. coli* BL21 (DE3) to obtain the heterologous expression strain BL/pET28a-MtrA, which facilitates robust MtrA protein expression (Additional file 1: Fig. S4). Following induction by 0.2 mM IPTG, bacteria were collected, washed, resuspended in a lysis buffer, and sonicated on ice. Soluble MtrA protein was purified by Ni–NTA agarose chromatography (Qiagen) according to the manufacturer’s instructions. The purified protein was quantified by Quick Start Bradford Dye Reagent (Bio-Rad) and stored at − 80 °C.

### Electrophoretic Mobility Shift Assays

EMSA was performed according to the instructions of the kit. PCR amplified DNA was labeled with Cy5 at the 5’ end to obtain the probe. The 20 μL reaction system was configured according to the instructions. Electrophoresis was carried out in a 6% non-denaturing polyacrylamide gel. Protein-DNA complexes and free DNA were separated due to the difference in mobility, and the electrophoresis was observed using a fluorescence imaging system.

### RNA preparation and real-time qRT-PCR analyses

Sample RNA extraction was performed using the TRIzol Reagent /RNeasy Mini Kit (Qiagen) kit. cDNA synthesis was performed according to the kit SPARK script II RT Plus Kit (With gDNA Eraser). qRT-PCR analysis was used to determine the transcript levels of various genes [38]. Transcription of housekeeping gene 16S *rRNA* was used as internal control. Each experiment was performed in triplicate. *S. avermitilis* MA-4680 (BA000030.4) as a reference genome.

### Transcriptome data analysis

Gene expression statistics were generated using known reference gene sequences and annotation files as a comprehensive database, employing sequence similarity comparison to determine the expression abundance of each protein-coding gene in every sample. The number of reads compared to the protein-coding genes on each sample was obtained using the htseq-count software. After obtaining counts, protein-coding genes were filtered to remove genes with zero reads counts. The expression of protein-coding genes was calculated using the FPKM method [38]. Differential expression analysis was performed using DESeq2 software, adjusted by Benjamini and Hochberg’s method for controlling false discovery rate [39]. The condition for screening DEGs was set as ∣log2 fold Change∣ > 1 and P < 0.05. GO and KEGG databases were enriched for DEGs [40, 41].

### Fermentation and HPLC analysis of avermectins

The spore-rich single colony was isolated by dilution plate method and transferred to YMS plate. It was then cultured at 28 ℃ for 5–7 days. After abundant gray spores had grown, they were transferred to the seed medium and cultured in a temperature-controlled shaker at 220 rpm for about 48 h at 28 ℃. Subsequently, the culture was inoculated into the fermentation medium with an inoculum amount of 5% and incubated in a small shaker at 28 ℃ for 10 days.

400 μL of *n*-butanol was added to 1 mL of fermentation broth, and the supernatant was centrifuged at 12,000 rpm for 10 min after vortexing and shaking for 1 h. The supernatant was filtered in the organic phase, and the yield of avermectin was determined by high performance liquid chromatography (HPLC). HPLC analysis conditions: a C18 reversed-phase column with a length of 150 mm and an inner diameter of 4.6 mm was used. The mobile phase consisted of a methanol–acetonitrile-ammonium acetate aqueous solution (40:55:5) at a flow rate of 0.8 mL/min, which was maintained throughout the analysis. The injection volume was 10 μL, the detection wavelength was 246 nm, and the running time was 15 min.

Transfer 1 mL of the fermentation broth into a centrifuge tube, and centrifuge at high speed to separate the supernatant. Retain the bacterial cells and wash them twice with distilled water. Centrifuge again at 12,000 rpm for 5 min, followed by drying in an oven until constant weight is achieved to determine the biomass of *Streptomyces* [42].

### Supplementary Information

**Additional file 1: Table S1.** Primers used in this study. **Table S2.** Transcriptional regulators with significant changes in transcript levels in DmtrA. **Table S3.** Other secondary metabolism-related genes with significantly altered transcript levels in DmtrA on day 2 and 6. **Fig. S1.** Deletion vector pKCDmtrA construction. **Fig. S2.** overexpression vector pIBOmtrA. **Fig. S3.** Complementary vector pSECmtrA. **Fig. S4.** pET28a-MtrA vector mapping (a) and colony PCR electropherogram (b) M: DL2000, 1 ~ 8: PCR validation of different monoclonal colonies. **Fig. S5.** MEME predicted MtrA binding motif. **Fig. S6.** GO enrichment of differentially expressed genes in WT and DmtrA strains on the second day and 6th day of fermentation. **Fig. S7.** KEGG enrichment of differentially expressed genes of WT and DmtrA strains on day 2 and day 6.

## Data Availability

All data supporting the conclusions of this article are included within the manuscript and additional file.

## Abbreviations

WT: Wide-type
OmtrA: *mtrA* Overexpressing strain
DmtrA: *mtrA* Deletion strain
C-DmtrA: *mtrA* Complementary strain
EMSA: Electrophoretic mobility shift assays
qRT-PCR: Quantitative RT-PCR
HPLC: High-performance liquid chromatography
HTH: Helix-turn-helix
PKSs: Polyketide synthases
REC: Phosphoacceptor receiver
DEGs: Differentially expressed genes
