# Supplementary material for: Characterization of a pleiotropic regulator MtrA in Streptomyces avermitilis controlling avermectin production and morphological differentiation
Source: Microb Cell Fact. 2024 Apr 8;23:103. doi: 10.1186/s12934-024-02331-2 (PMC11000389; doi:10.1186/s12934-024-02331-2)
Supplement: Supplementary file 1 — Additional file 1: Table S1. Primers used in this study. Table S2. Transcriptional regulators with significant changes in transcript levels in DmtrA. Table S3. Other secondary metabolism-related genes with significantly altered transcript levels in DmtrA on day 2 and 6. Fig. S1. Deletion vector pKCDmtrA construction. Fig. S2. overexpression vector pIBOmtrA. Fig. S3. Complementary vector pSECmtrA. Fig. S4. pET28a-MtrA vector mapping (a) and colony PCR electropherogram (b) M: DL2000, 1 ~ 8: PCR validation of different monoclonal colonies. Fig. S5. MEME predicted MtrA binding motif. Fig. S6. GO enrichment of differentially expressed genes in WT and DmtrA strains on the second day and 6th day of fermentation. Fig. S7. KEGG enrichment of differentially expressed genes of WT and DmtrA strains on day 2 and day 6. [file 12934_2024_2331_MOESM1_ESM.doc]

## Additional file

**Characterization of a pleiotropic regulator MtrA in *Streptomyces avermitilis* controlling avermectin production and morphological differentiation**

Jinpin Tian^1,#^, Yue Li^1,#^, Zhang Chuanbo^1, 2, 3^, Jianyu Su^4, 5*^, Lu Wenyu^1, 2, 3*^

^1^School of Chemical Engineering and Technology, Tianjin University, Tianjin, PR China.

^2^Frontiers Science Center for Synthetic Biology, Tianjin University, Tianjin, PR China.

^3^Key Laboratory of System Bioengineering (Tianjin University), Ministry of Education, Tianjin, PR China.

^4^Key Laboratory of the Ministry of Education for Conservation and Utilization of Special Biological Resources in the Western, Yinchuan 750021, China.

^5^College of Life Science, Ningxia University, Yinchuan 750021, Ningxia, China.

*Corresponding author: Jianyu Su, Email: [su_jy@nxu.edu.cn](mailto:su_jy@nxu.edu.cn)

*Corresponding author: Wenyu Lu, Email: [wenyulu@tju.edu.cn](mailto:wenyulu@tju.edu.cn)

# Jinpin Tian and Yue Li contribute equally to this article.

**Table S1** Primers used in this study

| **Primers** | **Sequence（5’-3’）** |
| --- | --- |
| m-left-F | CATGATTACGAATTCACCTGTGCTGGTCCTTCTCGACC |
| m-left-R | AGCCGCTGTACATGCTAAACGACATCATCCCATTAGC |
| m-right-F | GGATGATGTCGTTTAGCATGTACAGCGGCTGCGCTCCAAAG |
| m-right-R | GCCAGTGCCAAGCTTCCGCTCGGCGATCCCCGCAGCCATCC |
| pKC-F1 | ACGGTGTCGCTGAACGAGGCGTTCAAGG |
| pKC-R1 | GGTGAATTCGTAATCATGTCATAGCTGTTTCCTGTGTG |
| pKC-F2 | GAAGCTTGGCACTGGCCGTCGTTTTACAACGTCGTG |
| pKC-R2 | GTTCAGCGACACCGTCTGGTGCCGGTCCCGCCGG |
| 139-m-F | GGAATTCCATATGATGATGTCGTTTATGAAGGGACGAGTC |
| 139-m-R | CCGGAATTCTCAGCTCGGTCCGGCCTTGTAACCGAC |
| 152-m-F | TACGAATTCGATATCATGGCTGATCAGTACGCGC |
| 152-m-R | CAGGTCGACTCTAGATCAGCTCGGTCCGGCCTTG |
| pSET-F | GCCGGACCGAGCTGATCTAGAGTCGACCTGCAGCC |
| pSET-R | GTACTGATCAGCCATGATATCGAATTCGTAATCATG |
| Check-up-F | CAGCGAGTCAGTGAGCGAGGAAGCG |
| Check-down-R | TGCATCGGCCGCGCTCCCGATTCCGGAAG |
| Check-pKC-F | TAGGCCGAAGAGGCTCACGACGAGG |
| Check-pKC-R | CGAGTGCGTCGCGGAACGCGGCCTG |
| Check-Dm-F | CTGCACACCTTCAATTGTCCGCCGGATAC |
| Check-Dm-R | GATCTCCAGCAGATCCGCGAGCAGCGAC |
| Check-Om-F | GTAAAACGACGGCCAGTGCCAAGCTTGG |
| Check-Om-R | CACACAGGAAACAGCTATGACATGATTAC |
| Check-Cm-F1 | ACCGGATAAGGCGCAGCGGTCGGGCTG |
| Check-Cm-R1 | ACAGCGCCTGGGCGGCCTCGTCCACGTCG |
| Check-Cm-F2 | GCCCGGCCGGGACGGTATCGAGGTGTGC |
| Check-Cm-R2 | CAGCGTCTCCGACCTGATGCAGCTCTCG |
| MtrA-F | AGAAGGAGATATACCATGATGAGCTTCATGAAAGG |
| MtrA-R | GTGGTGGTGGTGCTCGAGGCTCGGACCTGCTTTG |
| pET28a-F | GAGCACCACCACCACCACCACTGAGATCCGGCTGC |
| pET28a-R | GGTATATCTCCTTCTTAAAGTTAAACAAAATTATTTC |
| Check-M-F | GGTGATGTCGGCGATATAGGCGCC |
| Check-M-R | ATCCGGATATAGTTCCTCCTTTCAGC |
| e-P*_aveR_*-F | AGCCAGTGGCGATAAGTACATACCGGTCACCCGGTATTCCATTC |
| **Primers** | **Sequence（5’-3’）** |
| e-P*_aveR_*-R | AGCCAGTGGCGATAAGCATTCCGCCCTCGGCGACGAGCAGTTTC |
| e-*aveD*_*aveA1*-F | AGCCAGTGGCGATAAGCCAGTATCCGAGGTGGGTGTTCC |
| e-*aveD*_*aveA1*-R | AGCCAGTGGCGATAAGCGGCTTCGCAGGCGCGGACGCTCTC |
| e-*aveA1_aveA2*-F | AGCCAGTGGCGATAAGAAGTCACTCATGTTGAGGTGGAAC |
| e-*aveA1_aveA2*-R | AGCCAGTGGCGATAAGGTGACGTCGCCCCGCCTGGGTAACG |
| e-*aveA4*_*orf1*-F | AGCCAGTGGCGATAAGTCCGGATCATAGAAAGCCTCCACG |
| e-*aveA4*_*orf1*-R | AGCCAGTGGCGATAAGGCCGGCCGATGAGGCGGCAACCATG |
| e-*aveBIII*_ave*BIV*-F | AGCCAGTGGCGATAAGTCACCAGGATGTCTTTTATGCCGCC |
| e-*aveBIII*_ave*BIV*-R | AGCCAGTGGCGATAACCGCCGTCAGGTCGGCCGTACGCGTC |
| e-P*_aveBVIII_*-F | AGCCAGTGGCGATAAGAGACCAGTCGCGTCGGAGCCTCCGC |
| e-P*_aveBVIII_*-R | AGCCAGTGGCGATAAGGACCGTTCCGAGCGCCAGTCGGCTG |
| e-P*_whiB_*-F | AGCCAGTGGCGATAAGGGGTGCGGTGGTTCCTCGGCGTGGG |
| e-P*_whiB_*-R | AGCCAGTGGCGATAAGGGGAAAGAAGGACTCGGGGTCGGT |
| e-P*_whiH_*-F | AGCCAGTGGCGATAAGAAGGGCGCTCGAAGGCCCGTCTGTT |
| e-P*_whiH_*-R | AGCCAGTGGCGATAAGCGCGGGAATGCCGACGCGGTCGGC |
| e-P*_ssgC_*-F | AGCCAGTGGCGATAAGCTACTCCTTGATGTTCCAGGGCATC |
| e-P*_ssgC_*-R | AGCCAGTGGCGATAAGGGACTCGCTCGACACAACGAGGCGC |
| e-P*_bldD_*-F | AGCCAGTGGCGATAAGGGCGAGGAAGACGCCTCGGGTCGGAATG |
| e-P*_bldD_*-R | AGCCAGTGGCGATAAGTTCTCCTCGACACCGTGGAGGGAAA |
| e-P*_bldM_*-F | AGCCAGTGGCGATAAGCCAGTCCCAGAGGTCCGCGTTCGGA |
| e-P*_bldM_*-R | AGCCAGTGGCGATAAGCTTCCTCGCCGTTGGCCGCCGTCGTG |
| real-aveR-F | AAAGAGTACGTTCCTGGGCG |
| real-aveR-R | TCAGCAGTTGCTGCAGTACG |
| real-aveF-F | TCTCGACTTCTGCGAACAGG |
| real-aveF-R | TTCTGGTGGCCGTTCACTAC |
| real-aveD-F | CTCAGCTTGCCGATGAGGAG |
| real-aveD-R | GTGGGGGACTACTACGACCG |
| real-aveA1-F | CTTCGACTCCGTCATGGGTG |
| real-aveA1-R | GTGGTGTGGTCGAAGATGAGC |
| real-aveA2-F | TGCGATCACTATCGACACCG |
| real-aveA2-R | GGTGAAGACGAAGGGAGTGG |
| real-aveC-F | TGTAGTAGACACCCGACCGTC |
| **Primers** | **Sequence（5’-3’）** |
| real-aveC-R | GCAACGACAGCAGTTCACG |
| real-aveE-F | CTCCAGGAATCAGCGGTCTC |
| real-aveE-R | CCATCCTCGTAGCCGTATCG |
| real-aveA3-F | AGTGTCATCGTCCTTGGCAG |
| real-aveA3-R | TGATGACCATCCACTCACGC |
| real-aveA4-F | GAGACCATCACCGCGAAGAG |
| real-aveA4-R | CTCGAACCGTACCTGGACTG |
| real-orf1-F | TCGTGGACCTGCTGGTGAAC |
| real-orf1-R | CGGCAACCATGAATGACACCCC |
| real-whiB-F | CGACCCCGAGTCCTTCTTTC |
| real-whiB-R | GTATTCGAGGCACTCGGAGC |
| real-bldD-F | GGGGAGCTATATGTCCAGCG |
| real-bldD-R | GACACCGTGGAGGGAAAGG |
| real-bldM-F | ATGACATCCGTCCTCGTCTG |
| real-bldM-R | GAGGACTTCCTCGCCGTTG |
| real-ssgC-F | GCATGTCGATTTCGCCGAC |
| real-ssgC-R | GTACAGGCAGTGAGGACTCG |
| real-olmA2-F | CTCACCGAGAACCGAGAGTG |
| real-olmA2-R | GTACGACATGTGCGTTGGTG |
| real-olmA4-F | GAGAACACCGGTCTCGATCC |
| real-olmA4-R | GTGCTCAGATAGCCCTCGAC |
| real-melC1-F | GATGGAACTGCACGTGATGC |
| real-melC1-R | TCAGTTGAAGGGGACGAGC |
| real-crtI-F | GCTCTGCACCTTCTCGGTG |
| real-crtI-R | GACCGGTGTCGATGAGGTAG |
| real-crtV-F | CATCCGATTCGCTGGGAGG |
| real-crtV-F | GTACCAGTTGCCCGATGGTC |
| real-pks3-2-F | CGAGGACATCCAGTACGTCG |
| real-pks3-2-R | CAGGAACTGCTTCAGGAGCC |
| real-pks9-1-F | GACCTGGTGGGGTACATACG |
| real-pks9-1-R | ATAGCCCATATCGGCGAACG |
| real-ptlI-F | ACTTCATGTCCTCGCTGTCC |
| real-ptlI-R | GCGGTCGTAGAACACTCCTC |
| real-ptlG-F | ACGGAATCACCTATGCGCTC |
| real-ptlG-F | ATGAGGAAGGTGACGTTCGG |
| 16s-F | CACGGAGAGTTTGATCCTGGC |
| 16s-R | CCAGAGTGCAGGGCAGATTG |

1. The underlined sequence is the cleavage site sequence

**Table S2** Transcriptional regulators with significant changes in transcript levels in DmtrA

| **Gene** | **Log_2_ (fold change)** | **Up or down regulation** | **Family** |
| --- | --- | --- | --- |
| *sav_6994* | -1.003 | Down | PadR-like family |
| *sav_2270* | -1.703 | Down | TetR-family |
| *sav_1829* | -1.736 | Down | ROK-family |
| *sav_5033* | -1.980 | Down | None |
| *sav_1699* | -3.695 | Down | MarR-family |
| *sav_3850* | -1.297 | Down | None |
| *sav_2240* | -3.334 | Down | None |
| *sav_3216* | -1.451 | Down | WhiB-family |
| *sav_6745* | -2.888 | Down | None |
| *sav_5312* | -2.199 | Down | LacI-family |
| *sav_4985* | -2.711 | Down | None |
| *sav_3701* | -2.798 | Down | IclR-family |
| *sav_5741* | -1.662 | Down | IclR-family |
| *sav_4189* | 2.387 | Up | MarR-family |
| *sav_2914* | 1.984 | Up | None |
| *sav_678* | 1.592 | Up | None |
| *sav_4782* | 4.103 | Up | TetR-family |
| *sav_5028* | 1.138 | Up | None |
| *sav_4281* | 1.706 | Up | MarR-family |
| *sav_2781* | 2.180 | Up | MerR-family |
| *sav_2012* | 1.608 | Up | None |
| *sav_1526* | 2.548 | Up | LysR-family |
| *sav_1739* | 3.330 | Up | GntR-family |
| *sav_3850* | 1.058 | Up | None |
| *sav_4023* | 1.244 | Up | GntR-family |
| *sav_2759* | 1.745 | Up | TetR-family |
| *sav_3568* | 1.017 | Up | TetR-family |
| *sav_5032* | 1.900 | Up | None |
| *sav_980* | 2.121 | Up | GntR-family |
| *sav_1263* | 1.730 | Up | TetR-family |
| *sav_3203* | 2.579 | Up | LuxR-family |
| *sav_5336* | 3.053 | Up | MarR-family |
| *sav_1476* | 1.623 | Up | ROK-family |
| *sav_2114* | 2.822 | Up | LacI-family |
| *sav_4448* | 1.124 | Up | MarR-family |

**Table S3** Other secondary metabolism-related genes with significantly altered transcript levels in DmtrA on day 2 and 6

| **Gene** | **Log_2_ (fold change)** | **Description** |
| --- | --- | --- |
| Non-ribosomal peptide-6 (nrp6) | | |
| *sav_601* | -1.231 | *fecD1*, putative ABC transporter iron (III)/siderophore permease protein |
| *sav_602* | -1.641 | *fecB*, putative ABC transporter iron(III)/siderophore-binding protein |
| Isorenieratene | | |
| *sav_1021* | 1.424 | *crtY*, lycopene cyclase |
| *sav_1023* | 3.391 | *crtI*, phytoene desaturase |
| *sav_1025* | 2.178 | *crtV*, putative methylesterase |
| Melanin cluster | | |
| *sav_1136* | 1.302 | *melC1*, tyrosinase co-factor protein |
| *sav_1137* | 1.228 | *melC2*, tyrosinase |
| γ-butyrolactone cluster | | |
| *sav_2270* | -1.703 | *avaL1*, putative TetR-family transcriptional regulator |
| Polyketide-3 cluster | | |
| *sav_2272* | -4.335 | hypothetical protein |
| Polyketide-3 cluster | | |
| *sav_2274* | -3.946 | putative secreted protein |
| *sav_2275* | -3.668 | putative transmembrane efflux protein |
| *sav_2276* | 1.534 | *fabH7,* putative 3-oxoacyl-ACP synthase III |
| *sav_2282* | -7.196 | *pks3-3*, putative acyl carrier protein |
| Oligomycin cluster | | |
| *sav_2890* | 2.134/-1.313 | *ccrA1*, crotonyl-CoA reductase |
| *sav_2892* | 2.711/-1.689 | *olmA4*, modular polyketide synthase |
| *sav_2893* | 2.630/-2.710 | *olmA5*, modular polyketide synthase |
| *sav_2894* | 2.254 | *olmB*, cytochrome P450 hydroxylase |
| *sav_2895* | 2.261/-1.644 | *olmA7*, modular polyketide synthase |
| *sav_2896* | 2.368/-1.431 | *olmA6*, modular polyketide synthase |
| *sav_2897* | 2.214/-2.248 | *olmA3*, modular polyketide synthase |
| *sav_2898* | 2.453/-2.106 | *olmA2*, modular polyketide synthase |
| *sav_2899* | 2.496/-2.284 | *olmA1*, modular polyketide synthase |
| *sav_2901* | 1.715 | *olmRII*, LuxR-family transcriptional regulator |
| Neopentalenolactone cluster | | |
| *sav_2991* | -2.063 | *ptlH*, alpha-ketoglutarate dependent hydroxylase |
| *sav_2992* | -1.011 | *ptlG*, putative transmembrane efflux protein |
| *sav_2993* | -1.834/-2.313 | *ptlF*, 1-deoxy-11beta-hydroxypentalenic acid dehydrogenase |
| *sav_2994* | -1.274 | *ptlE*, baeyer-villiger monooxygenase |
| Gene | Log_2_ (fold change) | Description |
| *sav_2995* | -1.588 | *ptlD*, putative dioxygenase |
| *sav_2997* | -1.834 | *ptlB*, farnesyl diphosphate synthase |
| *sav_2998* | -1.841 | *ptlA*, pentalenene synthase |
| *sav_2999* | -1.666/-1.639 | *ptlI*, cytochrome P450 |
| Aromatic polyketide | | |
| *sav_3665* | -3.694 | *pks8-8*, putative 3-oxoacyl-ACP synthase I |
| Polyketide-4 cluster | | |
| *sav_7184* | -4.364 | *pks4*, putative modular polyketide synthase |
| *sav_7186* | -6.787 | *cyp26*, cytochrome P450 hydroxylase |
| Polyhydroxycarboxylate siderophore cluster | | |
| *sav_7323* | 3.762 | *avsD*, putative diaminopimelate decarboxylase |

1. In the Log2 (fold change) column, the number in front of the slash indicates the change in transcript level on day 2 of fermentation, the number behind the slash indicates the change in transcript level on day 6 of fermentation, and those labeled with a single digit indicate that there is a significant difference in the transcript level of the gene only on day 2 or day 6 of fermentation.


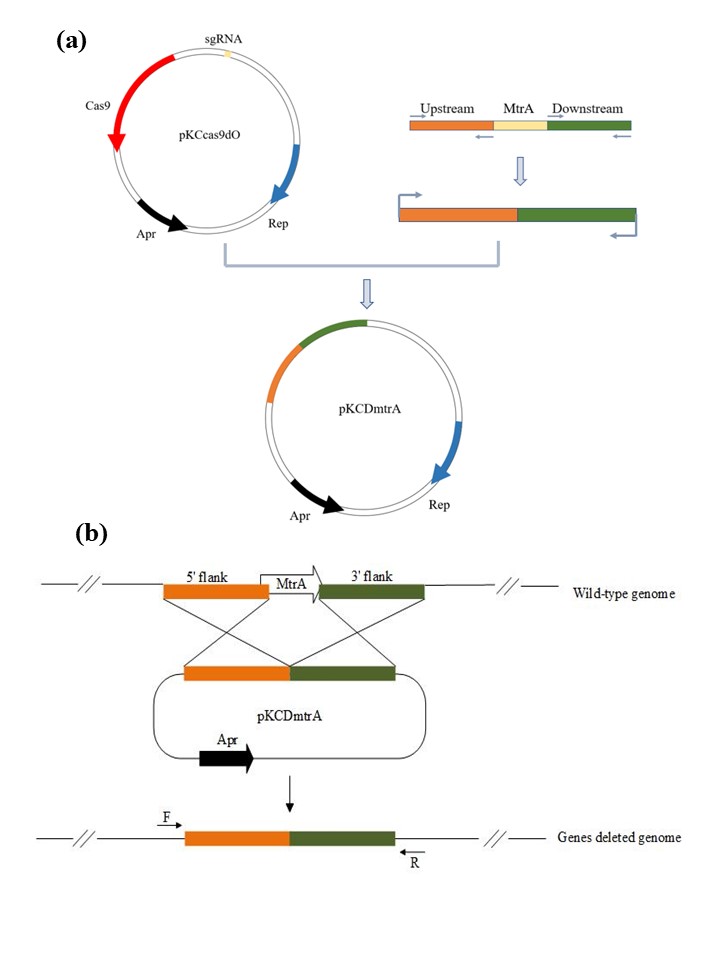


**Fig. S1** **Construction of deletion strains.** (a) Deletion vector pKCDmtrA construction. **(b)** Homologous recombination schematic of *mtrA* deletion.


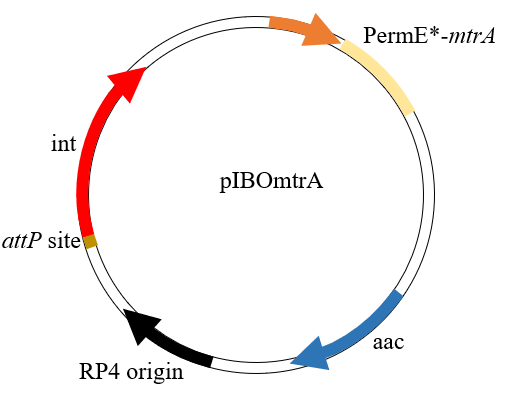


**Fig. S2** overexpression vector pIBOmtrA.


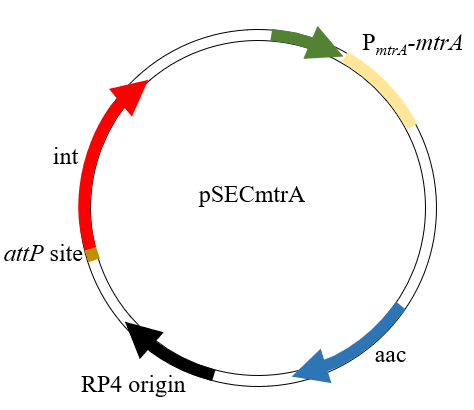


**Fig. S3** Complementary vector pSECmtrA.


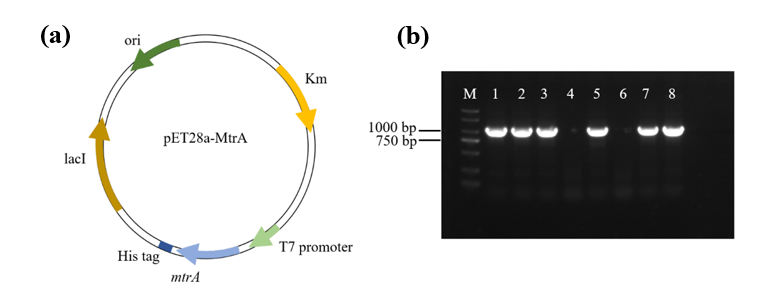


**Fig. S4** pET28a-MtrA vector mapping (a) and colony PCR electropherogram (b) M: DL2000, 1~8: PCR validation of different monoclonal colonies.


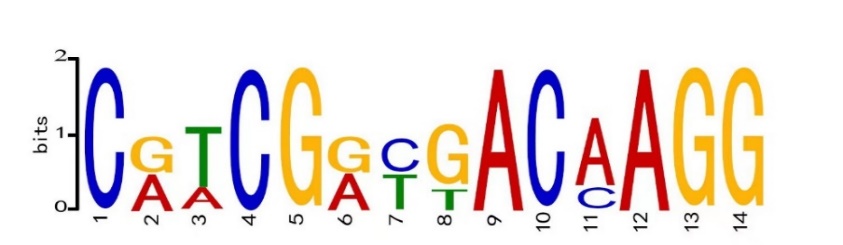


**Fig. S5** MEME predicted MtrA binding motif








**Fig. S6** GO enrichment of differentially expressed genes in WT and DmtrA strains on the second day and 6th day of fermentation








**Figure. S7** KEGG enrichment of differentially expressed genes of WT and DmtrA strains on day 2 and day 6
